# Morphology and transport characterization of solution-processed rubrene thin films on polymer-modified substrates

**DOI:** 10.1038/s41598-020-68293-8

**Published:** 2020-07-22

**Authors:** Xujing Gao, Wentao Liu, Hao Liu, Miaoming Huang, Suqin He, Manman Zhang, Zhengxia Hua, Chengshen Zhu

**Affiliations:** 1 School of Material Science and Engineering, Zhengzhou University, Zhengzhou, 450001 China; 2 Henan Key Laboratory of Advanced Nylon Materials and Application, Zhengzhou University, Zhengzhou, 450001 China

**Keywords:** Electronic devices, Atomic force microscopy

## Abstract

In this report, the morpho-structural peculiarities and the crystallization mechanisms in solution-processed, solvent vapor annealed (SVA) thin films of rubrene (5,6,11,12-tetraphenylnaphthacene) on different substrates were investigated. The high-quality rubrene crystal films with a triclinic crystal structure were successfully prepared on the FTO substrates (glass slide coated with fluorine-tin-oxide) modified by PLA (polylactic acid) for the first time. The area coverage of rubrene crystal and the sizes of rubrene dendritic crystals increased with increasing thickness of PLA film and concentration of rubrene solution. For rubrene molecules, FTO wafers with rough surface provided the possibility of heterogeneous nucleation. During the SVA process, there were two kinds of forces acting on the diffusion of rubrene molecules: one force was provided by the residual chloroform solvent, which was perpendicular to the substrate, and the other force was provided by gaseous dichloromethane, which was parallel to the substrate. The synergy of these two forces was proposed to explain the nucleation and the crystallization processes of rubrene films. The higher nucleus of PLA/rubrene dendrites and the layer-by-layer stacking of needle-shaped nanocrystalline PLA/rubrene were important for exploring their kinetic formation process.

## Introduction

Organic semiconductor materials, due to their outstanding performance in electronics and optoelectronics, have become the global focus for their promising potential in the research and applications of thin films and flexible electronic device^1-3^. Rubrene (5,6,11,12-tetraphenylnaphthacene), as a typical p-type organic semiconductor material, holds a very high record of field-effect mobility (20 cm^2^/V s)^4^ in the form of orthorhombic single crystal grown by physical vapor deposition method (PVD)^5–7^, and therefore is used in organic field-effect transistors (OFETs)^8^. Besides, rubrene single crystals can also be prepared by “hot wall” deposition method^9^, in situ vacuum annealing^10^, or solution growth methods (such as slow evaporation^11^ and cooling from supersaturated solution^12^). Rubrene thin films have been deposited by various techniques such as sol–gel coating^13^, combinatorial molecular beam epitaxy (MBE) technology^14^, solution-mediated vacuum deposition^15^, or spin-coating^16^. In initial works, OFETs based on rubrene thin-film demonstrated a very low mobility (~ 10^–6^ cm^2^/V s).^17^ Then, the mobility of polycrystalline rubrene OFET with hexamethyldisilazane (HMDS) coated SiO_2_ dielectric was found to reach up to 10^–2^ cm^2^/V s^18^. For the crystalline rubrene, considered as the active channel in OFETs, a variety of insulating organic polymers and small molecules have been used to induce crystallization of rubrene. Among these, the popular organics were 5, 12-diphenylanthracene and (ultra)high-molecular-weight polystyrene (UHMW-PS)^19^, poly(4-vinylpyridine) (P4VP) and poly(methyl methacrylate) (PMMA)^20^, 6,13-pentacenequinone (PQ)^21^, 6,13-diazapentacene (DAP)^22^ and so on. In addition to glass or silicon wafer, there are also several alternative substrates, such as highly doped silicon with a layer of SiO_2_^23^, SiO_2_ modified with different organic molecules^24–26^, indium tin oxide (ITO)^27^, ITO-PET (PET: polyethylene terephthalate)^28^, or octadecyltricholrosilane (OTS)-modified glass^29^. Among these studies, an efficient crystallization method of rubrene thin film with high charge carrier mobility (up to 3.5 cm^2^/V s)^30^ was reported, in which an adlayer was grown on a 5 nm-thick organic underlayer with optimum glass transition temperature by using thermal evaporation^31^.

Despite numerous investigations on rubrene crystals, it is still a challenge to prepare a large area thin film of rubrene crystals with high quality by cost-efficient processes. F-doped tin oxide (FTO) and ITO films with high conductivity and transparency to visible light are currently the most investigated and widely used conductive thin film electrodes, in particular for FTO due to its increased chemical stability^32,33^.

In this report, we used a simple spin-coating method to prepare rubrene films on FTO substrates and selected commonly used polymers (PS, PBSA (a blend of PBS (poly(butylene succinate)) and PBA (poly(butylene adipate))), and PLA (polylactic acid)) to induce the crystallization of rubrene via solvent vapor annealing.

## Methods

### Sample fabrication

Materials used in this work were purchased as follows: rubrene powder from Lingbao Central Plains Synthetic Materials Co., Ltd.; PBSA (3001MD) from Showa Denko, Japan; PS (molecular weight *M*_W_: 280,000) from Sigma-Aldrich; PLA (3052D) from NatureWorks, USA; PET (viscosity > 0.95 dL/g) from Yongsheng Xingda Plastics Co., Ltd., Urumqi, China.

**Figure 1:**
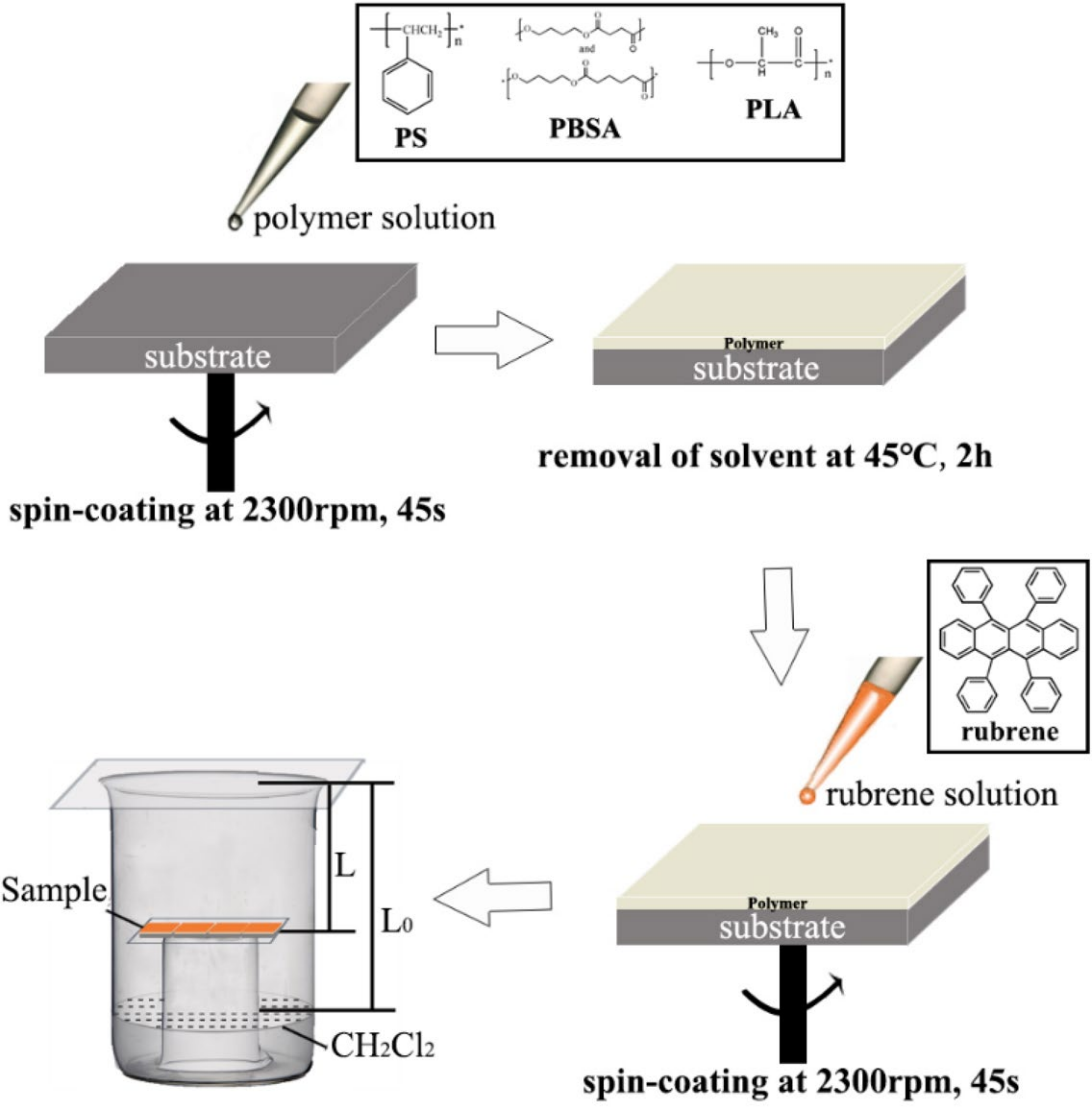
Schematic illustration of the fabrication procedure for polymer/rubrene crystal film.

The silicon (single-polished (100) lattice plane) and FTO (glass wafer coated with FTO, having a sheet resistance of less than 8 Ω/sq) wafers were successively sonicated in acetone and absolute ethyl alcohol, and then dried in a vacuum oven. The procedure of polymer/rubrene crystal film fabrication is illustrated in Fig. 1. First, a polymer layer was spin-coated on the bare substrate and was placed in a vacuum oven for 2 h at 45 °C in order to remove the chloroform solvent. Rubrene powder was dissolved in chloroform with different concentrations of 1, 3, 5, 7, and 9 mg/mL. Then, the rubrene solution was spin-coated on polymer thin film modified FTO wafers. In the last step, the polymer/rubrene films were fixed in an atmosphere of dichloromethane vapor (L/L_0_ = 0.4) for 24 h to obtain the crystal films.

### Measurements

The morpho-structural properties and the crystalline structure of the films were characterized by polarized optical microscopy (POM) (DM/LP, Leica, Germany), atomic force microscopy (AFM) (Multi Mode 8, Bruker, USA) operating in tapping mode under ambient conditions, and X-ray diffractometry (XRD) (UItima-IV, Rigaku, Japan).

The POM images were taken in transmission or reflection mode through two crossed polarizers. The crystal area coverage of the POM image was analyzed using ImageJ software. The AFM images were obtained using a rotated monolithic silicon probe (force constant of 40 N/m and a resonant frequency of 300 kHz, Tap 300 Al-G, Budget Sensors, Bulgaria). The XRD patterns were measured with an XRD diffractometer (Cu-Kα radiation, λ = 1.54 Å) under 40 kV and 30 mA tube current. The X-ray profile was recorded from 5 to 20° in steps of 0.02°, using automatic slits. The surface energies of substrates and polymer films were analyzed by contact angle measurements using water and ethylene glycol in conjunction with the Owens–Wendt–Rabel–Kaelble (OWRK) method^34^ (JC2000C1, POWEREACH, China).

### OFET fabrication and characterization

Bottom-gate and top-contact OFETs ware fabricated on a PET (polyethylene terephthalate) dielectric layer on the top of FTO gate electrode wafers to investigate the charge transport of PLA/rubrene thin film. The PET dielectric layer was fabricated by spin-coating the PET solution on the FTO wafer, and then was heated by placing the PET film modified FTO wafer on a 180 °C preheated hot plate. The small piece Au films (~ 100 nm thickness) were transferred on to PLA/rubrene thin film with the help of the mechanical probes, and then these thin Au layers were glued onto PLA/rubrene thin film by the Van der Waals force as the source and drain electrodes.^35^ The OFETs had the channel width of 217.1 μm and the channel length of 51.1 μm. The characterizations of the OFETs were carried out using a probe station (Semiconductor Characterization System 4,200 SCS, Keithley, USA) under ambient conditions. Field-effect mobility *μ* of PLA/rubrene was calculated using the following equation, *μ* = 2* k*/*C*_*i*_(W/L), where W and L are respectively the width and the length of the channel, *k* is the slope of the square root of the drain current versus gate voltage, and *C*_*i*_ is the areal capacitance of PET dielectric layer.

## Results and discussion

### The influence of substrates

**Figure 2:**
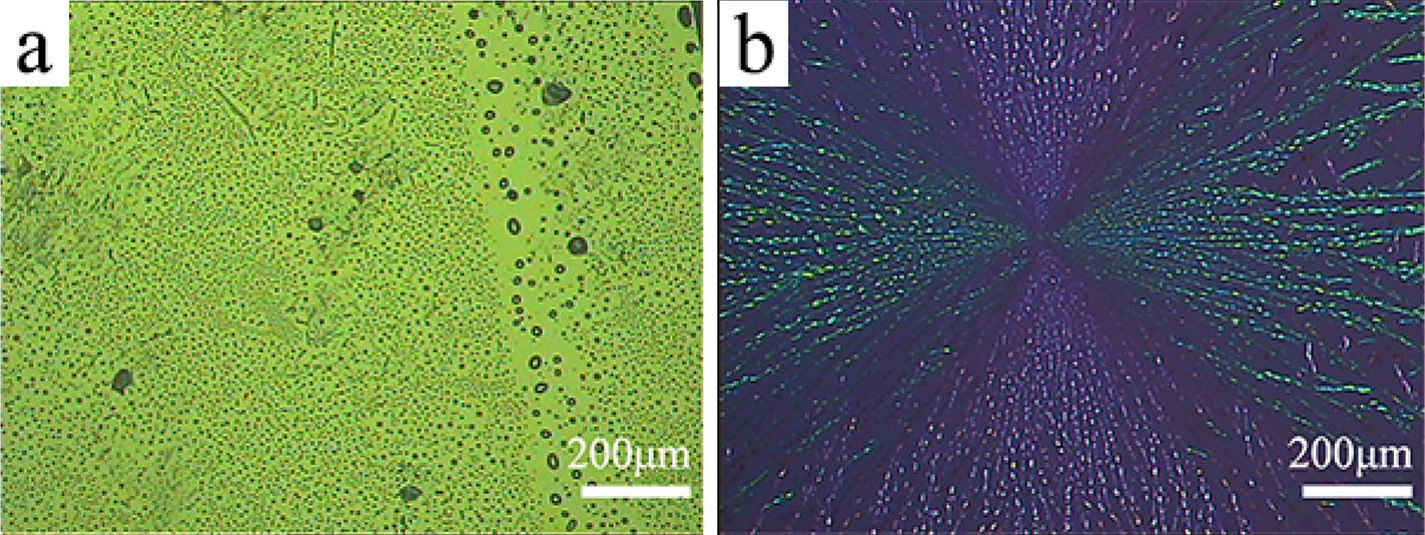
Polarized optical microscopy (POM) images of rubrene thin films obtained by spin-coating the rubrene solution on a silicon wafer (**a**), and a fluorine-doped tin-oxide (FTO) coated glass wafer (**b**) after the solvent vapor annealing procedure.

In Fig. 2, the surface morphologies of rubrene thin films on unmodified substrates are shown through POM images. It could be observed that the morphologies of rubrene films on the bare silicon wafer and FTO wafer are significantly different. In Fig. 2a, there are a dewetting phenomenon and some sporadic needle-shaped crystals on the silicon wafer and the needle-shaped crystals have a length of 30–150 μm, a width of ~ 1 μm. From the highlight shown in Fig. 2b, these crystal particles are directional and pervasive on the FTO wafer. Interestingly, the crystal film shows an alternating phenomenon of blue and green crystal domains on the POM image without a compensator. Although the surface energies of silicon and FTO are similar (Table S1), the surface roughness of the two wafers is different (Fig. S1). As shown in Table S2, the roughness *R*_*q*_ (Root mean square roughness) of silicon is only 0.49 nm, which is much lower than that of FTO (24.5 nm). In terms of the surface roughness, it is easier for rubrene to crystallize on the rough surface of the FTO wafer via the process of heterogeneous nucleation. In the later part of this paper, the investigated rubrene crystal films are obtained on FTO unless otherwise stated.

**Figure 3:**
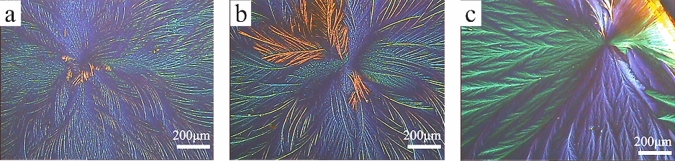
Polarized optical microscopy (POM) images of polymer/rubrene crystal films obtained by spin-coating the rubrene solution on a PBSA modified FTO wafer (**a**), a PS modified FTO wafer (**b**), and a PLA modified FTO wafer (**c**) after the solvent vapor annealing procedure. (The FTO wafers modified with polymer were fabricated by spin-coating the different polymer solutions on fluorine-doped tin-oxide (FTO) coated glass wafers, respectively.)

The continuity of rubrene crystal film is improved on FTO wafers modified by polymer, as illustrated in Fig. 3. In comparison with Fig. 2b, the rubrene crystal films become more continuous and denser on three different polymer films, especially on the PLA film (Fig. 3c). The surface morphology of the three polymer films on FTO substrates could be observed in Fig. S1. Furthermore, the surface roughness of the three polymer films is higher than that of bare FTO wafer (Table S2), so the higher surface roughness improves the heterogeneous nucleation of rubrene. Additionally, the surface energies of three polymer films are 41.5 $\pm$ 0.3, 33.1 $\pm$ 1.5, and 36.3 $\pm$ 0.7 mJ/m^2^, respectively (Table S1), so the crystalline rubrene having the surface energy of 33.9 mJ/m^2^^36^ is more well-fitted to the polymer than to FTO (47.6 $\pm$ 0.9 mJ/m^2^, in Table S1). Besides, the crystalline quality of a PS/rubrene crystal film or a PLA/rubrene crystal film is higher than that of a PBSA/rubrene crystal film, and this phenomenon may be related to the side benzene ring of PS or the side methyl of PLA.

**Figure 4:**
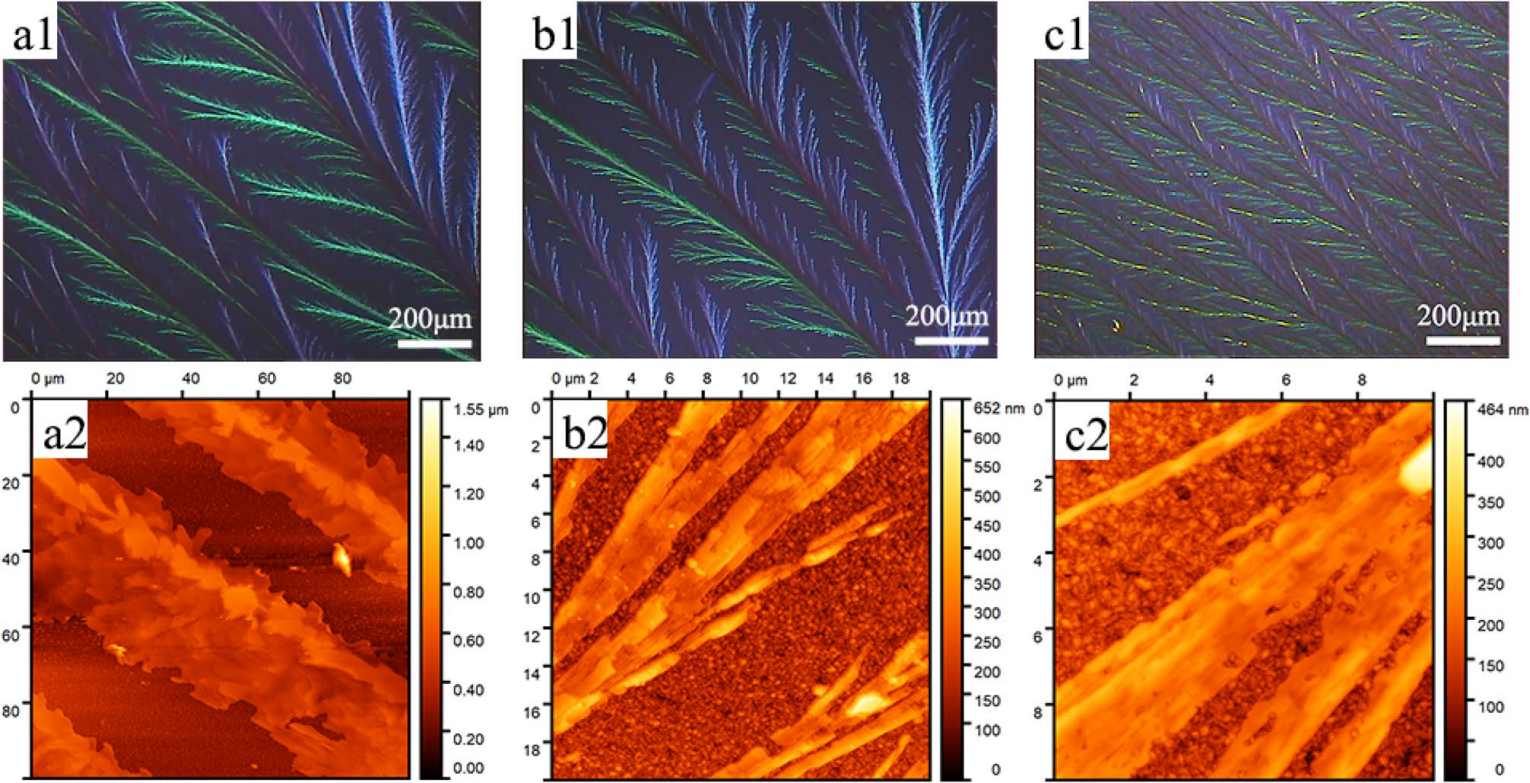
Polarized optical microscopy (POM) (**a1**, **b1**, **c1**) and atomic force microscopy (AFM) (**a2**, **b2**, **c2**) images of PLA/rubrene crystal films obtained by spin-coating the rubrene solution on a 12.6 nm PLA modified FTO wafer (**a1**, **a2**), a 22.2 nm PLA modified FTO wafer (**b1**, **b2**), and a 57.2 nm PLA modified FTO wafer (**c1**, **c2**) after the solvent vapor annealing procedure (the PLA films with different thickness were fabricated by spin-coating the different concentration PLA solutions on fluorine-doped tin-oxide (FTO) coated glass wafers, respectively).

In the next step, the rubrene crystal films on PLA films with different thicknesses were fabricated (Fig. 4). The thickness of PLA films could be observed in Fig. S2 and Table S3. With increasing thickness of PLA film, the rubrene crystal films become denser, and the lateral branches of rubrene dendrite also appear. In Fig. 4a2, the width of rubrene dendrite on a 12.6 nm PLA film is at least 20 μm, some as wide as 40 μm. The lateral branches could be more clearly observed in Fig. 4b2 and Fig. 4c2, where the width of lateral branches is also in the micron range. The increasing thickness of PLA film suggests that there are more PLA macromolecules involved in the heterogeneous nucleation of rubrene film, explaining the appearance of lateral branches. In contrast, PS had an impeditive effect betweeen the crystal nuclei and rubrene molecules.^37^ It can be supposed that PLA has a promoting effect owing to the good flexibility^38^ of chain segment, while the hindering effect of PS may be attributed to the weak flexibility because of the existence of the side benzene ring and π-π interactions^39,40^ between the side benzene ring and rubrene molecules.

### The influence of rubrene concentration

**Figure 5:**
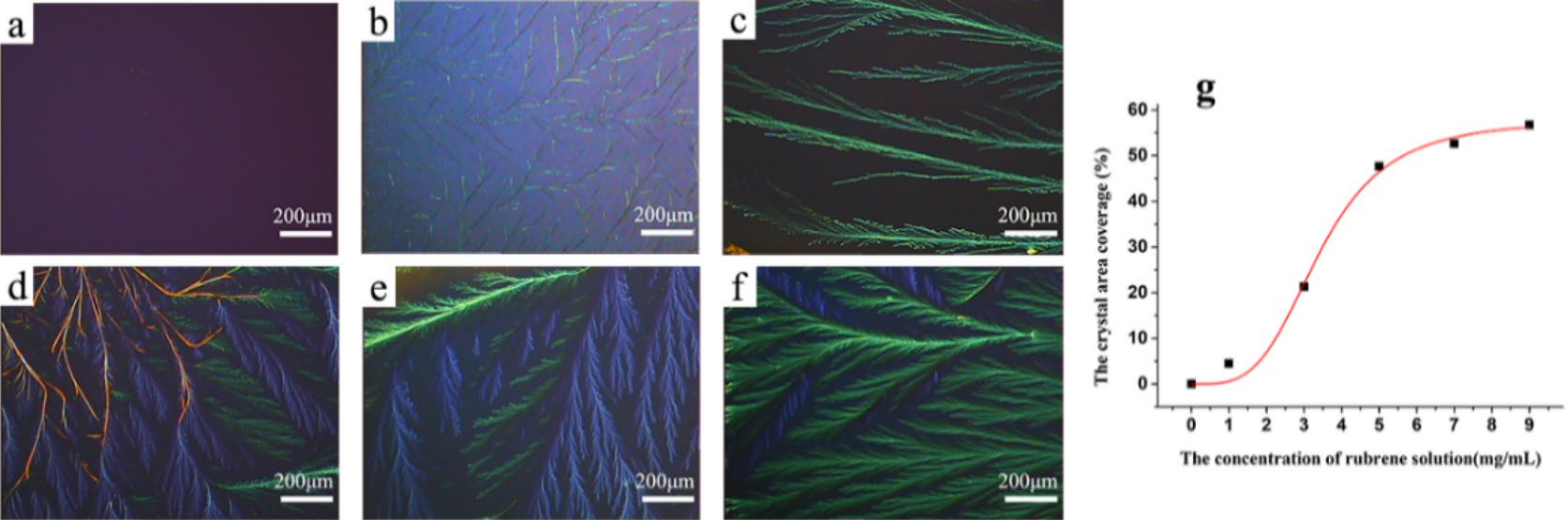
Polarized optical microscopy (POM) images (**a**–**f**) and the crystal area coverage (**g**) of PLA/rubrene crystal films obtained by spin-coating different concentration rubrene solutions on 57.2 nm PLA modified FTO wafers after the solvent vapor annealing procedure (the 57.2 nm PLA modified FTO wafers were fabricated by spin-coating the PLA solutions on fluorine-doped tin-oxide (FTO) coated glass wafers, and the concentrations of rubrene solutions were **a** 0, **b** 1, **c** 3, **d** 5, **e** 7, and **f** 9 mg/mL, respectively).

**Figure 6:**
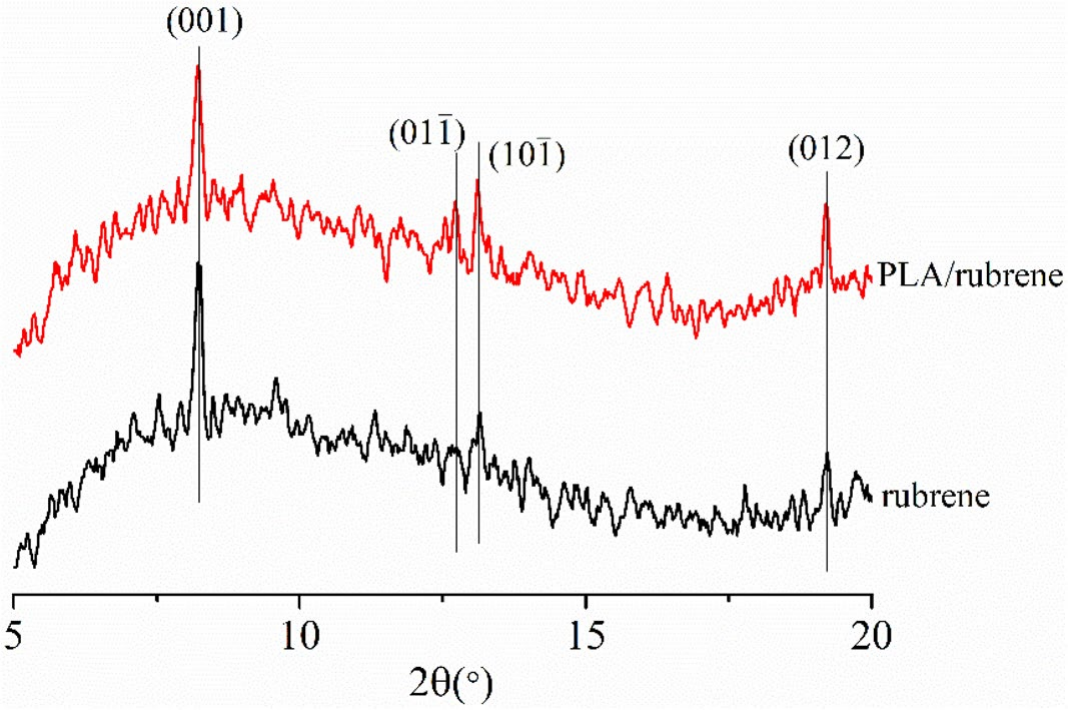
X-ray diffraction (XRD) patterns of rubrene crystal films obtained by spin-coating the rubrene solution on a fluorine-doped tin-oxide (FTO) coated glass wafer (black line), and a 57.2 nm PLA modified FTO wafer (red line) after the solvent vapor annealing procedure.

The influence of rubrene concentration on the morphology of rubrene crystal film was also investigated. The rubrene solutions with various concentrations were spin-coated on 57.2 nm PLA modified FTO wafers, postprocessing with SVA. The morphology of as-obtained PLA/rubrene crystal films could be observed in Fig. 5b–f. The original PLA film is amorphous (Fig. 5a), whereas the PLA/rubrene films exhibit dendritic crystallization (Fig. 5b–f). With the increase of rubrene concentration, the PLA/ rubrene crystal films become denser, the length of the main branch is up to centimeter-sized, and the crystal area coverage of dendrite increases obviously. In Fig. S3, it is more intuitive that the crystal areas vary along with the rubrene concentration. The POM images were then analyzed using Image-J software, and the crystal area coverage of PLA/rubrene films is shown in Fig. 5g. The coverage of dendrite has a noticeable increase and is up to 48% when rubrene concentration is 5 mg/mL, and the ultimate crystal area coverage is stable at around 55%. XRD was carried out to confirm the crystal structure of rubrene films, and the results are shown in Fig. 6. Single crystal rubrene has a triclinic structure with unit cell parameters of a = 0.70 nm, b = 0.85 nm, and c = 1.20 nm^41^. In Fig. 6, the peak at 2θ = 8.22° is assigned to an interplanar distance of 1.08 nm, which can be attributed to the (001) crystal plane of the triclinic rubrene crystal. Also, the other diffraction peaks of the triclinic rubrene crystal are discerned in Fig. 6.

### Crystallization mechanism of rubrene crystal films

**Figure 7:**
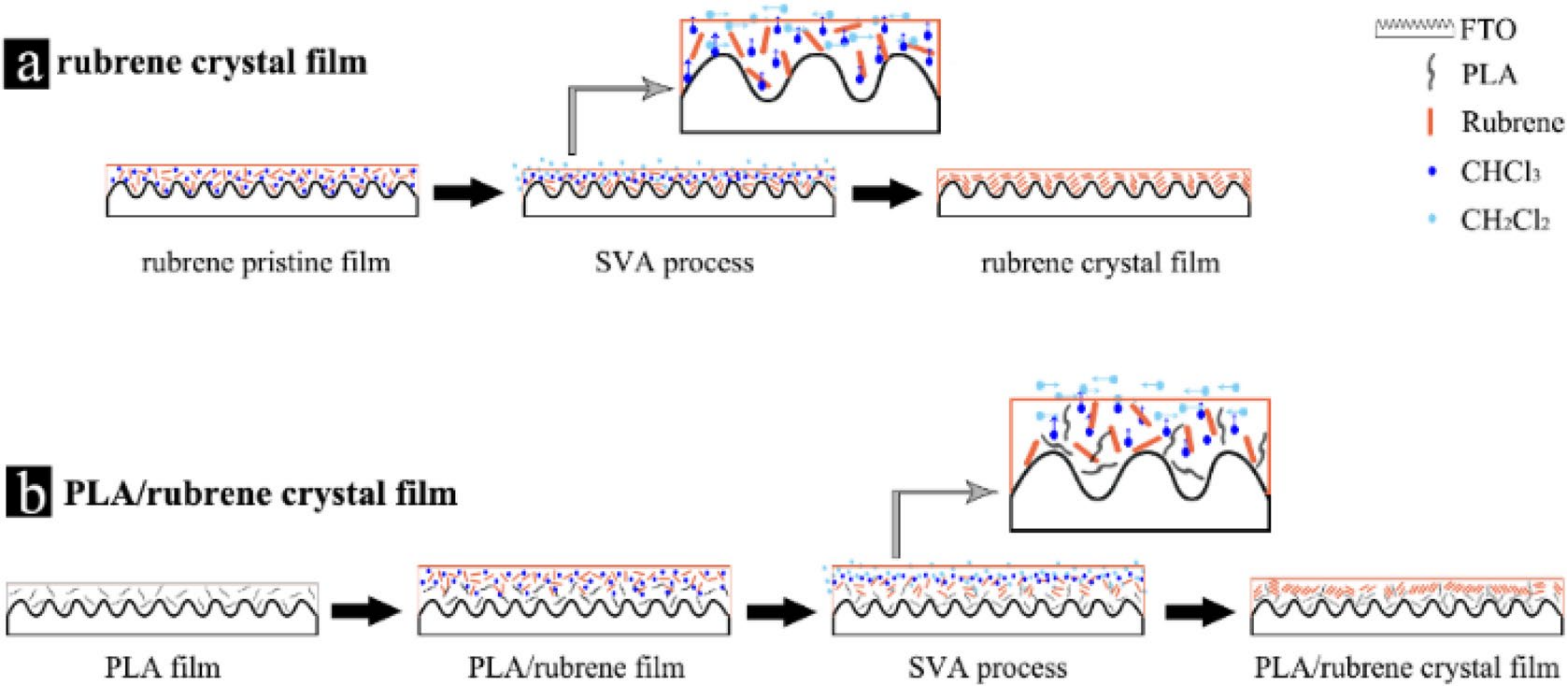
Schematic illustrations of the crystal mechanisms of the two films during the solvent vapor annealing process.

According to the previous results, the crystallization mechanisms are suggested for the as-obtained rubrene films (Fig. 7). Compared with the flat silicon wafer, it is beneficial for the heterogeneous nucleation of rubrene molecules on an uneven FTO substrate. Following the spin-coating process, the solvent chloroform molecules are not efficiently removed from the rubrene pristine film or PLA/rubrene film, and the residual solvent molecules provided the perpendicular molecular mobility to the substrate for rubrene. During the SVA process, the gaseous dichloromethane molecules penetrated into rubrene pristine film, and the rubrene molecules can diffuse in the direction parallel to the substrate (Fig. 7a). Ultimately, the crystal nuclei surrounds the convex part on the surface of FTO substrate, and the rubrene crystal film is formed. In the case of PLA/rubrene film, there are some PLA macromolecules dissolved in rubrene film on the surface of PLA film (Fig. 7b). Thus, the existence of PLA macromolecules makes rubrene molecules easier to nucleate.

**Figure 8:**
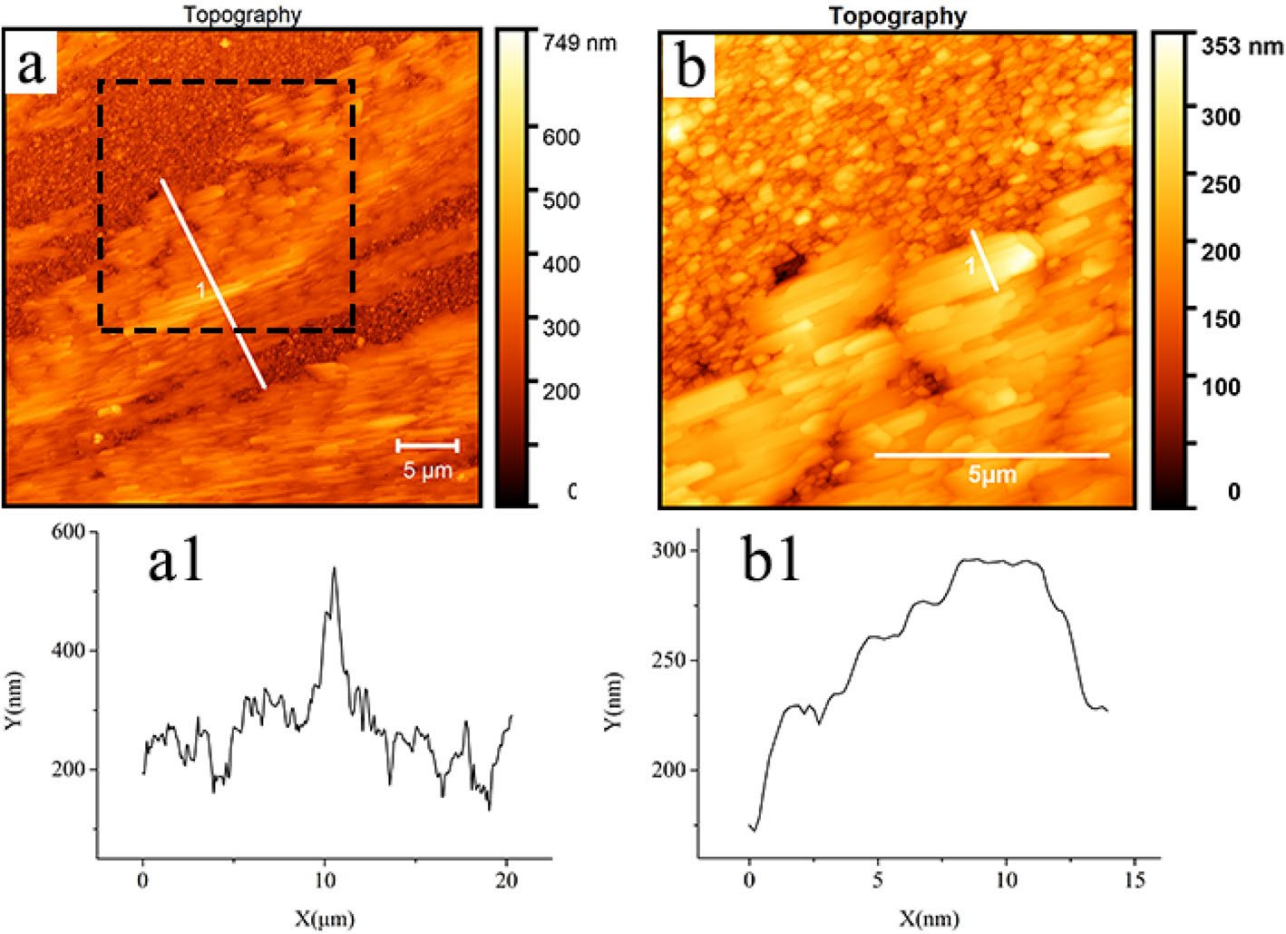
Atomic force microscopy (AFM) images of PLA/rubrene crystal film obtained by spin-coating the 10 mg/mL rubrene solutions on a 57.2 nm PLA modified FTO wafer after the solvent vapor annealing procedure.

In order to investigate the crystallization mechanism from the microscopic view, AFM images of PLA/rubrene film are shown in Fig. 8. Figure 8b shows a magnified height morphology of the section (marked by dashed rectangles) in Fig. 8a. The structural characteristics of dendrites in Fig. 8a are in accordance with that in Fig. 5b–f. Importantly, the magnified AFM image (Fig. 8b) of PLA/rubrene dendrites shows that they are composed of needle-shaped nanostructures. Moreover, the stacking of PLA/rubrene aggregates is parallel to each other. The height profile of PLA/rubrene dendrites (Fig. 8a1) shows that the height of the nucleus region is slightly higher (ca. 200 nm) than that of the other crystal region. Besides, the height profile (Fig. 8b1) shows that the needle-shaped nanocrystalline PLA/rubrene is step-growth. Thus, it can be affirmed that these nanocrystalline PLA/rubrene are stacked layer by layer. The high nucleus of PLA/rubrene dendrites and the layer-by-layer stacking of the needle-shaped nanocrystalline PLA/rubrene are important for exploring their kinetic formation process.

**Figure 9:**
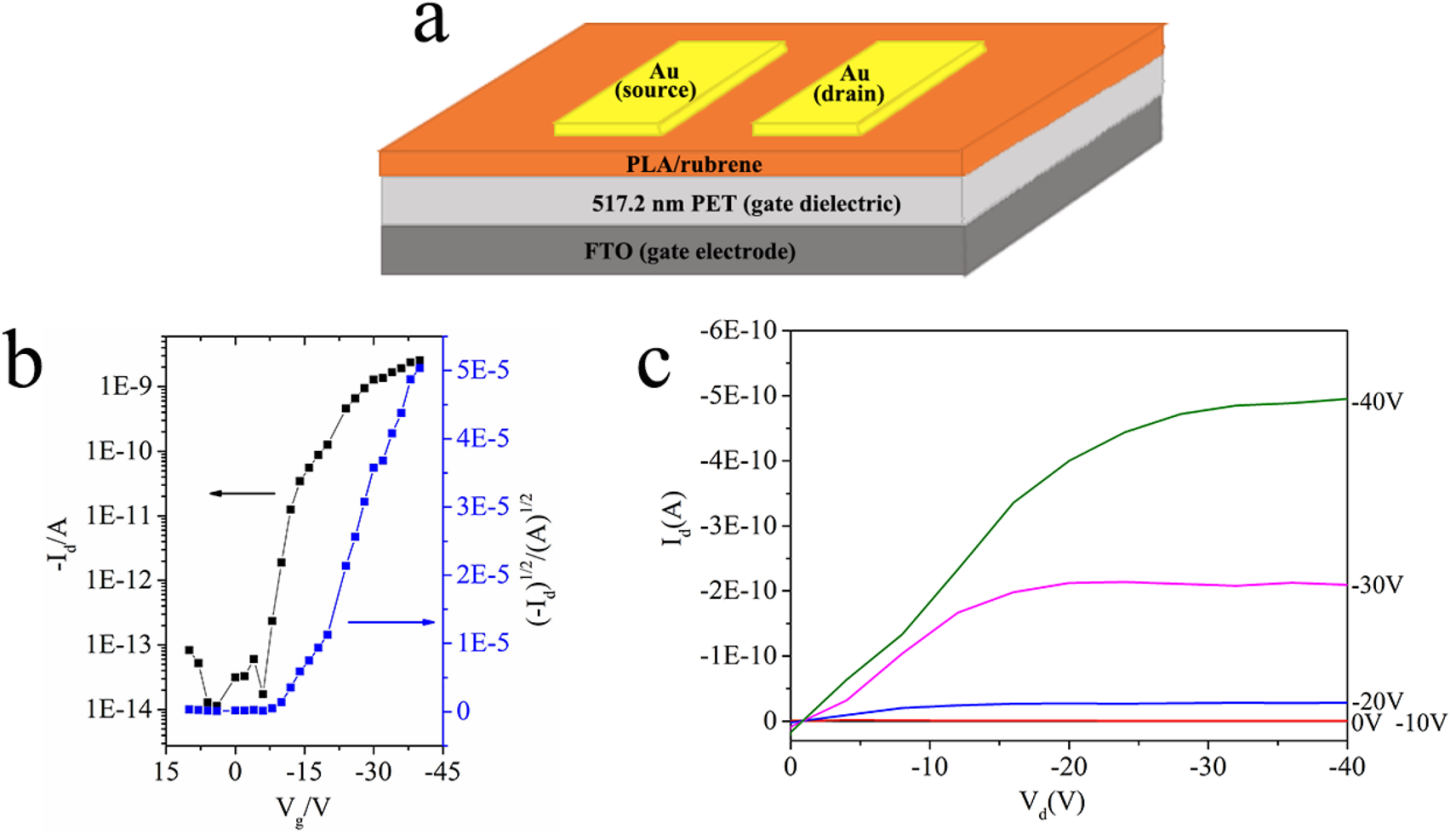
(**a**) Schematic diagram of a bottom-gate and top-contact organic field effect transistor (OFET) based on PLA/rubrene thin film. The typical transfer characteristics (**b**) and output characteristics (**c**) of OFET based on PLA/rubrene thin film. PLA/rubrene thin film was fabricated by successively spin-coating the 5 mg/mL PLA solution and the 10 mg/mL rubrene solution on a 517.2 nm PET modified FTO wafer after the solvent vapor annealing procedure, and the PET (areal capacitance *C*_*i*_ = 6.5 nF/cm^2^) was used as the gate dielectric of OFET.

Finally, the charge transport of PLA/rubrene thin films was investigated by fabricating bottom-gate and top-contact OFETs on the FTO wafers with a PET gate dielectric layer (Fig. 9a). The average thickness of the PET film can be observed in Fig. S4. The transfer characteristics of OFET based on PLA/rubrene thin film (Fig. 9b) shows a typical p-type semiconducting behavior. Calculated using the following equation, *μ* = 2* k*/*C*_*i*_(W/L), the field-effect mobility of PLA/rubrene is 2.68 $\times$ 10^–4^ cm^2^/V s, and the on/off ratio of the OFET is 10^5^.

## Conclusion

In the present work, rubrene was spin-coated on different substrates (silicon wafer, FTO, and FTO modified by different polymer films (PBSA, PS, or PLA)). Rubrene crystal thin films were obtained on a rough FTO wafer or on polymer-modified FTO wafers. When the high-concentration rubrene solutions were spin-coated on FTO modified by a 57.2 nm PLA film, the large area rubrene crystal films with a triclinic system formed. The coverage of the dendrites increased, even up to 48% when the concentration of rubrene solution was 5 mg/mL, and the ultimate crystal area coverage was stable at around 55%. OFETs based on PLA/rubrene crystal films were constructed on a PET dielectric layer on the top of FTO gate electrode wafers, and the carrier mobility of PLA/rubrene crystal film was 2.68 $\times$ 10^–4^ cm^2^/V s. Although the carrier mobility of PLA/rubrene is not high due to its triclinic system form, the addition of PLA macromolecules is a research point for the crystallization of rubrene. The crystallization of rubrene thin film was attributed to the unevenness of the FTO surface and the increase of PLA macromolecules engaged in the heterogeneous nucleation of rubrene. During the SVA process, a synergic process between the perpendicular mobility to the substrate provided by the residual chloroform solvent and the parallel mobility provided by gaseous dichloromethane on the diffusion of rubrene molecules was proposed to explain the nucleation and the crystallization mechanisms of the rubrene films. The formation mechanism of rubrene crystal film could be inferred from the higher nucleus of PLA/rubrene dendrites and the layer-by-layer stacking of needle-shaped nanocrystalline PLA/rubrene.

## Supplementary information

Supplementary file1 (PDF 588 kb)

## Data Availability

All data generated or analysed during this study are included in this published article (and its Supplementary Information files).
