# Supplementary material for: Morphology and transport characterization of solution-processed rubrene thin films on polymer-modified substrates
Source: Sci Rep. 2020 Jul 22;10:12183. doi: 10.1038/s41598-020-68293-8 (PMC7376014; doi:10.1038/s41598-020-68293-8)
Supplement: Supplementary file 1 — Supplementary file1 (PDF 588 kb) [file 41598_2020_68293_MOESM1_ESM.pdf]

Supplementary Information

Morphology and transport characterization of solution-processed rubrene thin films on polymer-modified substrates

Xujing Gao [1], Wentao Liu [1,*], Hao Liu [1,*], Miaoming Huang [1,*], Suqin He[1,2], Manman Zhang [1], Zhengxia Hua [1], and Chengshen Zhu[1,]

1 School of material science and engineering, Zhengzhou University, Zhengzhou 450001, China

2 Henan Key Laboratory of Advanced Nylon Materials and Application, Zhengzhou University, Zhengzhou, 450001, China.

*Correspondence: wtliu@zzu.edu.cn; hliu@zzu.edu.cn; mmhuang@zzu.edu.cn (F.L.) +86-0371-67781590;

Table S1. Contact angle and surface energy of silicon, FTO and FTO/polymer film

	Contact angle (°)		Surface energy γ_{SV} (mJ/m^2)
	Water θ_1	Ethylene glycol θ_2	
silicon	52.5	40.8	48.1 $\pm$ 1.5
FTO	59	36.4	47.6 $\pm$ 0.9
FTO/PBSA	69.7	46.9	41.5 $\pm$ 0.3
FTO/PS	90.4	65.6	33.1 $\pm$ 1.5
FTO/PLA	76.2	55.4	36.3 $\pm$ 0.7

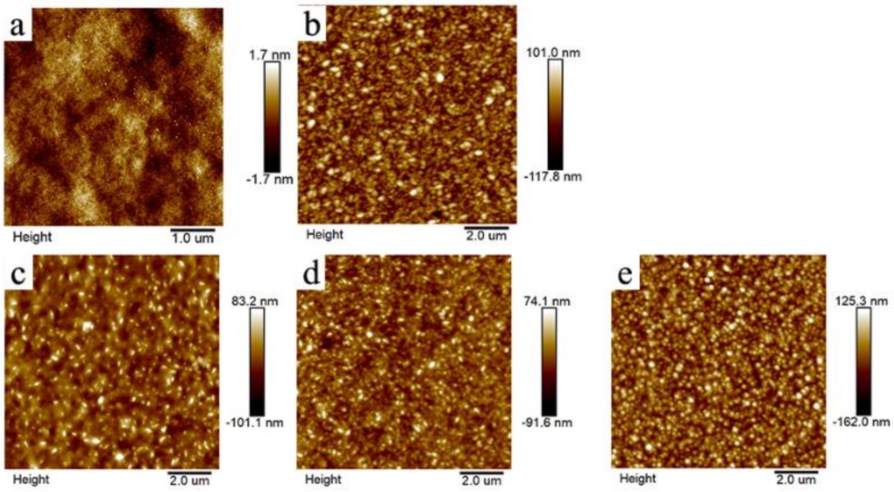

Figure S1. Atomic force microscopy images of FTO and FTO/polymer film (a. Si, b. FTO, c. FTO/PBSA, d. FTO/PS, e. FTO/PLA.)

Table S2. The surface roughness of different substrates

	Image R_q (nm)	Image R_a (nm)
Si	0.49	0.38
FTO	24.5	19.4
FTO/PBSA	30.9	24.1
FTO/PS	26.9	23.5
FTO/PLA	31.9	25.3

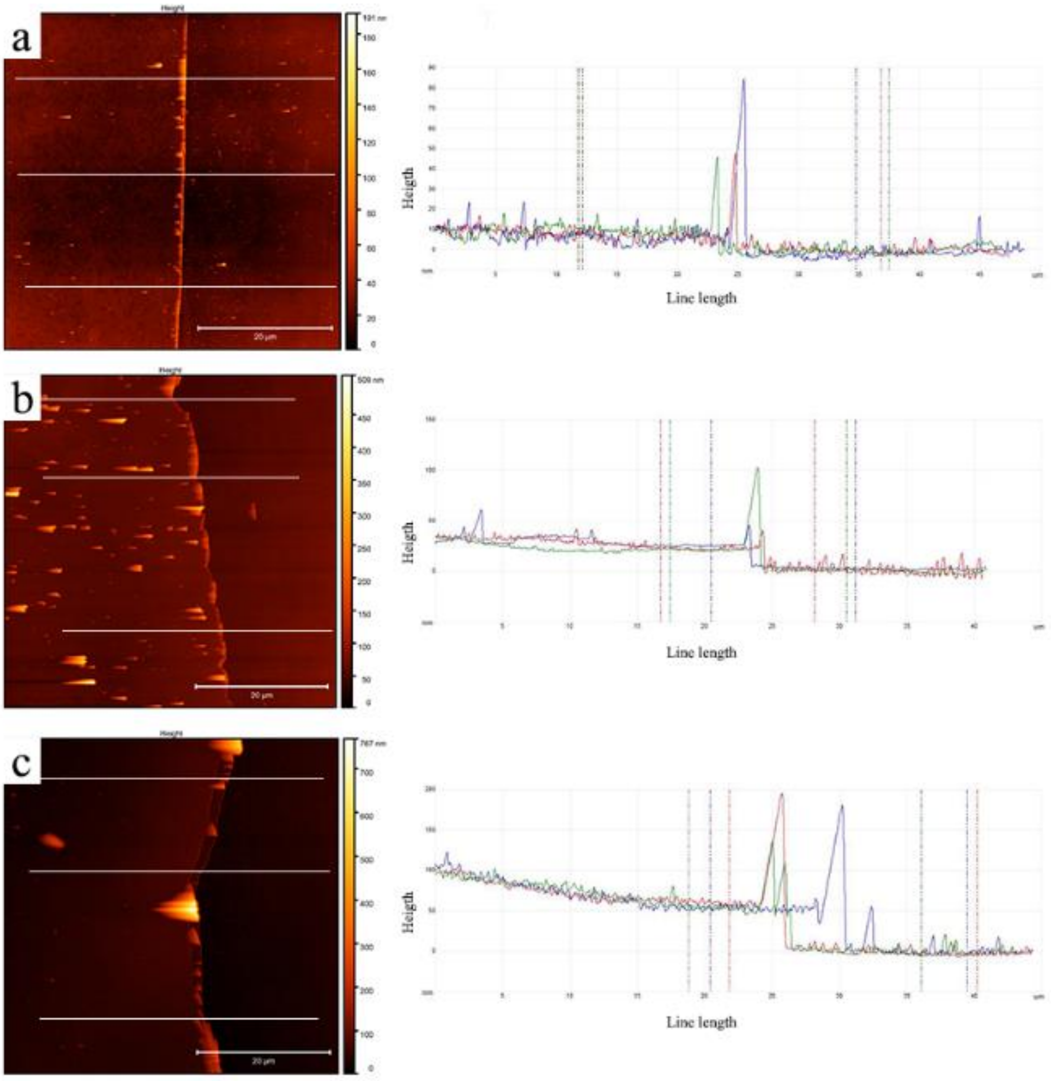

Figure S2. Atomic force microscopy images of PLA films on silicon substrates (PLA concentrations are a. 1, b. 3 and c. 5 mg/mL, respectively.)

Table S3. The thicknesses of PLA films for PLA solution with different concentrations

Solution concentration (mg/mL)	Film average thickness (nm)
1	12.6
3	22.2
5	57.2

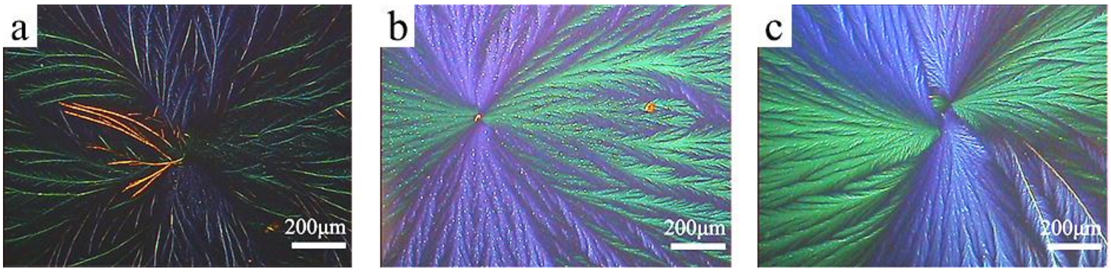

Figure S3. Polarized optical microscopy images of the central regions of PLA/rubrene crystal films fabricated by spin-coating different concentration rubrene solutions on 57.2 nm PLA modified FTO wafers after the solvent vapor annealing procedure. (Rubrene concentrations are a. 3, b. 5 and c. 9 mg/ml, respectively.)

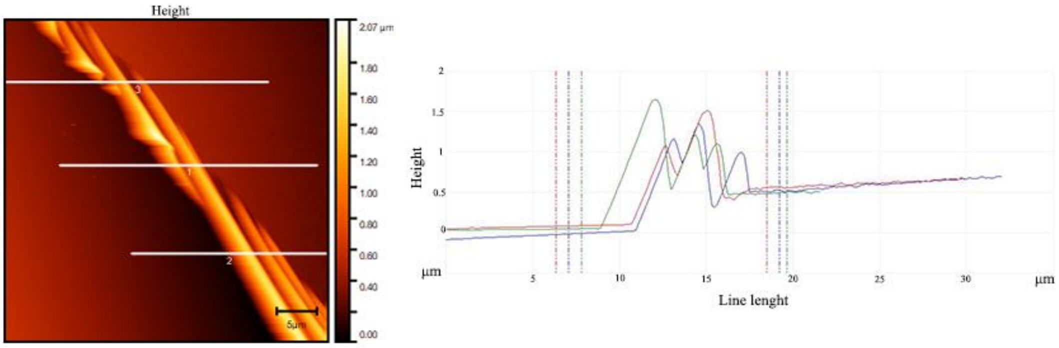

Figure S4. Atomic force microscopy image of PET films on a silicon substrate, the average thickness of PET film is 517.2 nm.
